# Is Methionyl-tRNA Synthetase Applicable as a Diagnostic Marker for Lung Cancer in Bronchial Ultrasound-Guided Brushing Cells?

**DOI:** 10.3390/diagnostics11101830

**Published:** 2021-10-03

**Authors:** Kyungjong Lee, Mijung Oh, Kyo-Sun Lee, Yoon Jin Cha, Yoon Soo Chang

**Affiliations:** 1 Samsung Medical Center, Department of Medicine, Division of Pulmonary and Critical Care Medicine, Sungkyunkwan University School of Medicine, Seoul 06351, Korea; kj2011.lee@samsung.com; 2 Medical Research Center, Sungkyunkwan University School of Medicine, Seoul 06351, Korea; mijung.oh1@gmail.com; 3 Department of Internal Medicine, Yonsei University College of Medicine, Seoul 06229, Korea; januscity@naver.com; 4 Department of Pathology, Yonsei University College of Medicine, Seoul 06229, Korea; yooncha@yuhs.ac

**Keywords:** aminoacyl-tRNA synthetase, aminoacyl-tRNA synthetase interacting multi-functional protein 2, aminoacyl-tRNA synthetase interacting multi-functional protein 2-exon 2 deletion, lung cancer

## Abstract

Background and objective: Methionyl-tRNA synthetase (MARS) and A variant of Aminoacyl-tRNA synthetase interacting multifunctional protein 2 (AIMP2) with an exon 2 deletion (AIMP2-DX2) are known to be overexpressed in lung cancer. However, their role as diagnostic markers in lung cancer has not been well established. Thus, we evaluated their diagnostic performance in brushed cells obtained from nodular lung lesions suspected of lung cancer. Methods: Samples obtained by radial endobronchial ultrasound-guided brushing were processed for cytological examination with Papanicolaou (Pap) staining. Then, double IF staining with MARS and AIMP2-DX2 antibodies was measured in the cytology samples for peripheral lung nodules. The diagnostic performance was compared against biomarkers. Results: MARS IF staining was the only independent staining method used for the prediction of malignant cells. The area under the curve (AUC) of conventional cytology, MARS IF, and MARS IF plus cytology was 0.64, 0.68, and 0.69, respectively. The diagnostic accuracy was increased in MARS IF plus conventional cytology compared with cytology alone (71% vs. 47%). Conclusions: The combination of MARS staining with conventional cytology showed increases in the diagnostic accuracy for diagnosing lung nodules suspected of lung cancer on chest-computed tomography scans.

## 1. Introduction

Lung cancer is the leading cause of cancer-related death in South Korea, although the 5-year survival rate has increased from 11.3% to 25.1% [1]. Early stage detection for surgical resection is crucial to improve the lung cancer survival rate. Lung cancer screening with low-dose computed tomography (LDCT) scanning enables early detection, and shows a survival benefit compared with a chest X-ray [2]. Thus, lung cancer screening with LDCT increases the detection of lung nodules needed to determine the malignancy of a lesion. In the era of lung cancer screening with LDCT, the acquisition of adequate tissue from lung nodules is essential for characterization. Radial endobronchial ultrasound (R-EBUS), used for the diagnosis of lung nodules, is a long, thin ultrasound probe providing a 360° view of the surrounding lung tissue [3]. Several additional sampling techniques improve the diagnostic yields of R-EBUS-guided lung biopsies, including brush cytology of the target lesion. However, these are not satisfactory due to their low additional diagnostic yields. For instance, the diagnostic yield of brush cytology is 34.8%, and this needs to be higher for biopsies of peripheral lung lesions [4]. Using cytology with Papanicolaou (Pap) staining methods for analysis may make it difficult to differentiate benign from malignant cells, which should be based only on cell morphology without consideration of cell architecture and nearby structures.

Aminoacyl-tRNA synthetases (ARSs) are housekeeping enzymes that catalyze protein synthesis. Recent studies have suggested that ARSs may not only catalyze the ligation of amino acids to their cognate tRNAs, but may also be associated with tumorigenesis [5]. Of the various ARSs, leucyl-tRNA synthetase (LRS), threonyl-tRNA synthetase, methionyl-tRNA synthetase (MARS), and glutamyl-prolyl-tRNA synthetase interact with proteins in the mTORC1, GCN2, CDK4, and VEGFR signaling pathways that play critical roles in cancer growth [6]. MARS transfers methionine to the initiator tRNA, which commences protein synthesis. MARS overexpression was shown to be evident in human colon cancer patients [7]; MARS may thus be involved in oncogenic transformation. However, it remains unclear whether MARS acts as an oncogenic driver, or whether oncogenic transformation reflects increased protein synthesis in cancer cells [8]. In terms of lung cancer, a recent study reported that patients with non-small-cell lung carcinomas (NSCLCs) exhibited MARS overexpression in tumor cells, and that poor clinical outcomes were closely associated with the MARS staining intensity and proportion [9].

Such findings suggest that MARS may be a useful diagnostic marker, especially given that it is detected incidentally during lung cancer screening. Additionally, immunofluorescence (IF) assays with MARS may facilitate the identification of atypical cells, which are difficult to interpret via Pap staining.

Aminoacyl-tRNA synthetase interacting multifunctional protein 2 exon 2 deletion variant (AIMP2-DX2) is overexpressed through alternative splicing in human lung cancer cells, and correlates with lung cancer stage [10]. Thus, ARS complexes, especially MARS and AIMP2-DX2, may hold potential as diagnostic markers in lung cancer patients.

In this study, we evaluated whether cytology IF staining using an MARS/AIMP2-DX2 antibody is useful for the detection of cancer cells from the brush cytology of suspected lung cancer patients.

## 2. Patients and Methods

### 2.1. Participant Enrollment

Patients with lung nodules on chest CT were prospectively enrolled in this study from August 2018 to November 2020. Patients were included if they met the following criteria: being at least 18 years of age with no history of other malignancy or NSCLC; having a chest CT indicating possible lung cancer; lung nodules more than 1 cm in diameter; being scheduled for R-EBUS-guided transbronchial lung biopsy (TBLB); and being eligible for needle aspiration, surgery, or clinical follow-up for confirmation of lung nodules. Patients who had metastases from other malignancies, or who received cancer-related treatment, were excluded.

### 2.2. Procedure and Equipment for Cytology Samples

R-EBUS-guided TBLB was performed under conscious sedation induced with midazolam and fentanyl if no endobronchial lesion existed. A 4 mm bronchoscope (BF P260F; Olympus, Tokyo, Japan) was used to reach into the suspected tumor following the guidance of computed tomography (CT) images. Then, the R-EBUS probe (1.4 mm, 20 MHz, UM S20-17S; Olympus) was inserted through the bronchoscope working channel. Once the target was located on ultrasound, R-EBUS-guided TBLB and bronchial brushing were performed through the working channel of the bronchoscope. Brushed cell material was alternatively rinsed in ThinPrep solution for cytology evaluation. All biopsy samples were sent to a pathologist for clinical diagnosis, and the liquid-based cytology slides were subjected to Pap and IF MARS and AIMP2-DX2 staining.

### 2.3. Definition of Diagnostic Classification

First, a primary classification of malignant or non-malignant was made based on the biopsy obtained by R-EBUS. A malignant diagnosis was confirmed in the case of primary lung cancer on R-EBUS-guided biopsy, and was designated as true-positive. Non-malignant outcomes, including a few atypical cells, fibrosis, and non-specific inflammation, were considered as indeterminate results. These patients were evaluated with other diagnostic efforts to obtain a final diagnosis, and the patients ultimately diagnosed with malignancy were considered false negative. A benign diagnosis including infectious diseases caused by a specific etiology and lesions that markedly improved in follow-up images were considered true negative. The final diagnosis remained unknown if all other diagnostic procedures were non-diagnostic, and no change was observed in follow-up images. Cytology specimens from brushing were classified into four categories: negative for malignancy, atypical cells, suspicious for malignancy, and positive for malignancy. Cytology results reported as negative for malignancy, containing atypical cells, or uncertain regarding malignancy were classified as benign or indeterminate results. In the case of results reported as positive for malignancy, malignant cytology was considered.

### 2.4. Antibodies and Cells

Anti-MARS antibody [EPR9873(B)] was obtained from Neomix (Cat #NMS-01-0003; Suwon, Korea) and Abcam (Cat# ab137105; Cambridge, UK), anti-AIMP2-DX2 rabbit monoclonal primary antibody was obtained from Molecular Medicine and Biopharmaceutical Science, Seoul National University. Anti-mouse-AF555 (#4409) and anti-rabbit-AF (#4412) were obtained from Cell Signaling technology (Danvers, MA, USA). MOLT-4, Daudi, and H460 cells were obtained from Korean Cell Line Bank (Seoul, Korea). ThinPrep PreservCyt^®^ was obtained from Hologic Inc. (#70097-002; Marlborough, MA, USA) and the Envision kit and 3,3′-diaminobenzidine (DAB) were obtained from Dako (#K3468; Carpinteria, CA, USA).

### 2.5. Reference Samples and Cell Lines

MARS and AIMP2-DX2 expression in a lung cancer cell line (NCI-H460 cells) and a lymphocyte cell line (Molt-4 and Daudi cells) were prepared as ThinPrep slides. These were used as positive and negative controls after IHC.

### 2.6. Immunofluorescence Staining

The ThinPrep slides were soaked in 1 × PBS for 5 min and then permeated with 0.2% PBS-T at room temperature for 30 min. After washing with 1 × PBS, the sections were blocked with 2% goat serum for 1 h and incubated with a primary antibody mixture diluted 1:250 with PBS for 90 min. After washing, sections were incubated with a 1:1000 diluted secondary antibody mixture containing anti-rabbit-AF488 and anti-mouse-AF555 for 1 h at room temperature and 4′,6-diamidole-2′-phenylindole dihydrochloride (DAPI) for 1 min to counterstain the nuclei. A Carl Zeiss LSM 750 confocal microscope was used to observe the immunofluorescence-stained specimens, and IF images were analyzed using ZEN lite software (Carl Zeiss). After triple staining with MARS and AIMP2-DX2 plus DAPI, the double staining of MARS+ and AIMP2-DX2+ in the cytoplasm was judged to be cancer cells. The expression level of MARS and AIMP2-DX2 staining was quantitatively indicated as 0 (absent), 1 (weak), 2 (moderate), or 3 (strong), and defined as cancer cells when the intensity score more greater than 2.

### 2.7. Statistical Analysis

Data are presented as number (%) for categorical variables and median (range) for continuous variables. Continuous variables were compared using the Mann-Whitney test, and categorical variables were compared using the Chi-square test. Logistic regression analysis was used to identify independent factors for diagnosing lung cancer. The sensitivity and specificity of the MARS/AIMP2-DX2 immunofluorescence cytologic staining, as well as the routine cytology report from the receiver operating characteristic (ROC) curve analysis, were used to determine the area under the curve (AUC) to compare the performances of the different diagnostic methods.

### 2.8. Ethics

This study was carried out in compliance with the Declaration of Helsinki and the Korean Good Clinical Practice guidelines, and was approved by the Institutional Review Board (IRB) of Samsung Seoul Hospital (IRB No. 2018-04-141-007). All patients provided written informed consent before enrollment.

## 3. Results

This study included 113 patients who underwent R-EBUS-guided lung biopsy because of indeterminate lung nodules on chest CT. Baseline characteristics are presented in Table 1. Among the enrolled patients, 87 had lung cancer and 26 were non-malignant cases. The median nodule size was 19.5 (14.0–27.0) mm in benign nodules and 28.0 (21.0–36.0) mm in malignant nodules. Malignant lung nodules comprised adenocarcinoma (77.0%), squamous cell carcinoma (18.4%), and non-small cell lung cancer (NOS; 3.4%) according to the histology. Benign lung nodules included organizing or other pneumonia (30.8%), nontuberculous mycobacteria infection (19.2%), aspergilloma (11.5%), mucoid impaction (7.7%), tuberculosis (7.7%), and other causes (cryptococcus, granuloma, sarcoidosis, pneumoconiosis, etc.). In terms of conventional cytology, especially in malignant lung nodules, interpretation as positive for malignancy was reported in only 31.0% of cases, whereas the other malignant lung nodules were interpreted as negative for malignancy (24.1%), atypical cells (35.6%), and suspicious for malignancy (9.2%) in conventional cytology, respectively.

In each cell, the characteristics of nuclear and cellular features were also described to find the differences between benign and malignant cytology. The nucleus diameter, presence of nucleoli, and cluster patterns of cells on slides were included. The nucleus diameter was larger in the malignant than the non-malignant cells, and there was a significant difference in the cluster patterns between the two groups. In about 80% of the cells diagnosed as lung cancer, three or more were clustered (Table 2).

**Figure 1:**
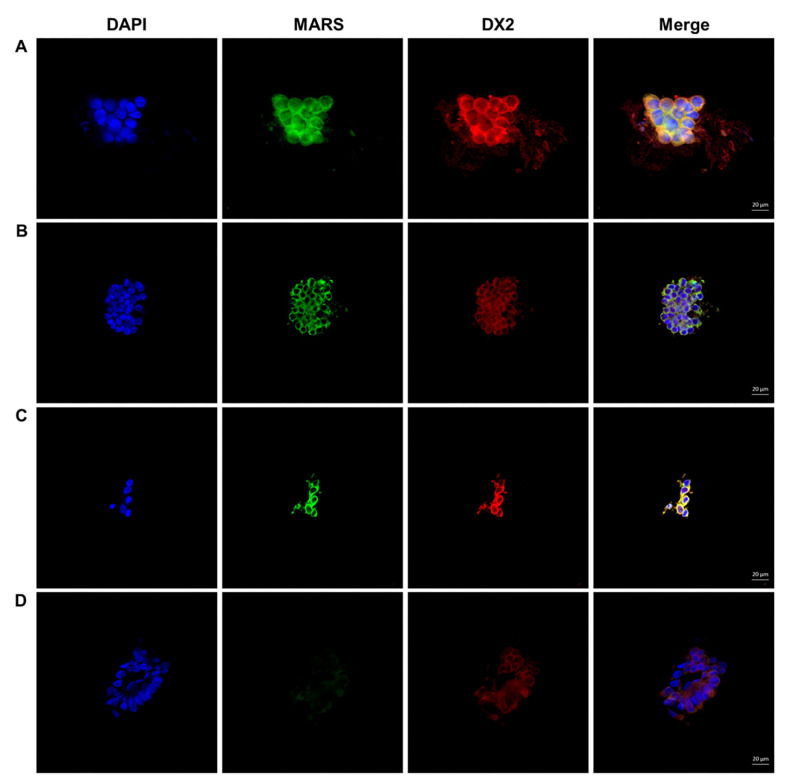
Immunofluorescence staining of MARS and AIMP2-DX2 in cancerous and benign cells. (**A**,**B**) Adenocarcinoma cases exhibiting intense cytoplasmic MARS and AIMP2-DX2 IF staining; (**C**) A Squamous cell carcinoma case evidencing intense cytoplasmic MARS and AIMP2-DX2 IF staining; (**D**) Benign cells exhibiting no or weak MARS and AIMP2-DX2 IF staining. DAPI: nuclear staining with 4′,6-diamidino-2-phenylindole; MARS: MARS IF staining; DX2: AIMP2-DX2 IF staining; Merge: combined images of MARS, AIMP2-DX2, and DAPI staining. Scale bar, 20 μm.

The double staining of MARS and AIMP2-DX2 was performed in the brush cytology samples, and interpreted as lung cancer in cases with moderate to strong staining of MARS and AIMP2-DX2. Weak or absent staining was regarded as a non-malignant nodule (Figure 1).

Univariable and multivariable analysis using logistic regression was performed to identify the best factors and combinations, based on cytological features and staining methods, to predict lung cancer. Univariable analysis showed that MARS, AIMP2-DX2, and double staining were the best predictors of cancer cells. Nucleus diameter and cluster pattern were also related to cancer cell prediction. However, multivariable analysis revealed that MARS staining was the only significant factor for diagnosing cancer cells (adjusted odds ratio (OR): 6.02, 95% confidence interval (CI): 1.32–28.09) (Table 3).

MARS and AIMP2-DX2 staining improved diagnostic sensitivity for malignant cells compared to conventional cytology alone. The sensitivity, specificity, and diagnostic accuracy of conventional cytology were 31%, 100%, and 47%, respectively. By contrast, the sensitivity, specificity, and accuracy of the staining method were 75%, 46%, and 68% for AIMP2-DX2 staining and 70%, 65%, and 69% for MARS staining, respectively. Adding MARS staining with conventional cytology improved the diagnostic performance to 72% for sensitivity, 65% for specificity, and 71% for diagnostic accuracy (Table 4). The sensitivity of MARS staining was greater for adenocarcinoma than squamous carcinoma (75% vs. 50%), while there was no significant difference in the MARS staining of cancer cells according to lung cancer stage.

**Figure 2:**
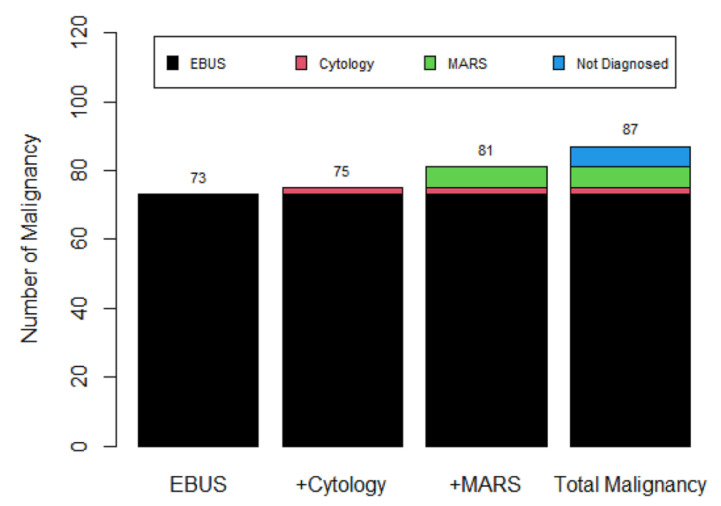
Additional diagnostic yield for malignancy compared to that from R-EBUS-guided TBLB alone. “EBUS” (left-hand bar) represents the number of patients with confirmed lung malignancies after only R-EBUS-guided TBLB. “Total malignancy” (right end bar) represents all malignant cases. Six cases (not diagnosed) among the total malignant cases were confirmed to be malignancies in other ways (for example, via surgical lung resection).

The diagnostic yield of R-EBUS-guided TBLB in terms of malignancy was 83.9% (73/87). Only two additional cases were added after analysis of the cytological results. However, the positive MARS staining of (confirmed) malignant lung nodules revealed six additional cases among patients negative for both R-EUBS-guided TBLB and conventional cytology. This increased the malignancy diagnostic yield from 83.9% to 93.1% (Figure 2).

**Figure 3:**
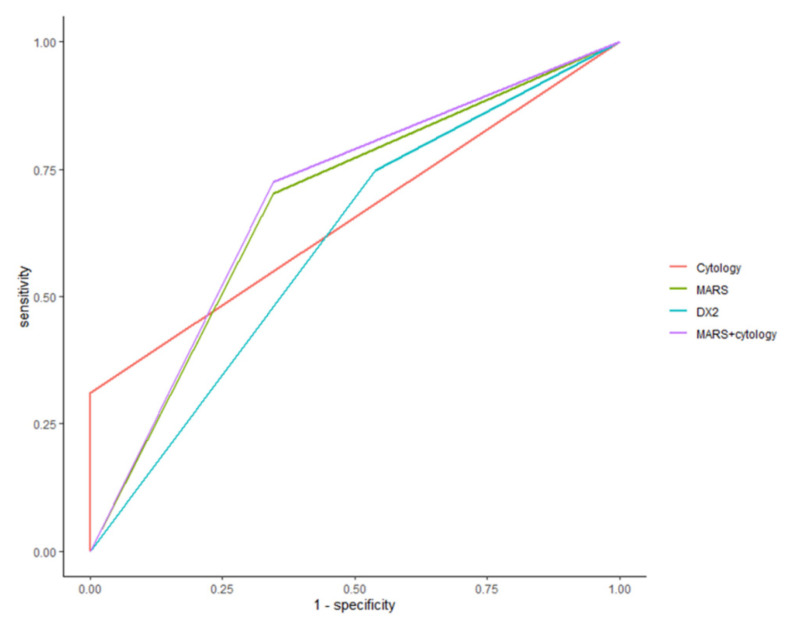
ROC for diagnostic performance according to the test methods. Cytology represents conventional cytology.

To determine the best performance, we calculated the AUC according to each diagnostic method. The ROC AUC from cytology, AIMP2-DX2, and MARS was 0.64 (95% CI: 0.58–0.70), 0.60 (95% CI: 0.50–0.71), and 0.68 (95% CI: 0.57–0.78), respectively. MARS staining with cytology showed an ROC AUC of 0.69 (95% CI: 0.58–0.79). Figure 3 shows the ROC AUC comparison for the different staining methods.

## 4. Discussion

We prospectively evaluated the diagnostic performance of MARS and AIMP2-DX2 IF staining, as well as conventional cytology, in 113 patients with lung nodules on chest CT. The combination of MARS and conventional cytology had greater sensitivity (73%) than conventional cytology alone (31%). The incidental discovery of lung nodules on chest CT is a common phenomenon in a clinical setting, and diagnosing the nature of the lung nodules on chest CT is problematic. R-EBUS-guided TBLB has been developed to obtain lung tissues through the bronchus. Bronchial brush cytology is usually performed following TBLB to increase the diagnostic yield. However, conventional cytology with Pap staining has a low diagnostic accuracy in differentiating benign and malignant cells because a visual interpretation is used. Thus, many cases are reported as atypical cells or suspicious. In some cases, malignant cells are interpreted as negative for malignancy if the cytology shows no specific features of cancer cells. Therefore, the development of new diagnostic staining methods to improve the diagnostic performance in the cytology from lung nodules is warranted.

ARSs comprise an enzymatic group that catalyzes the covalent amino acids to their complementary tRNA for protein synthesis. Among them, MARS has been reported to be overexpressed in human lung cancer and several other cancers [9,11]. MARS has housekeeping functions for initiating translation, but is also associated with tumorigenesis [12]. MARS may be overexpressed in several types of cancers as chromosome 12q13 locus with MARS, and CHOP is amplified in these tumors. This results in their overexpression and may play a role in tumor progression [13].

Three AIMPs serve as scaffold proteins for nine different ARSs to form a macromolecular complex [14]. Among AIMPs, ARS-interacting multifunctional protein 2 has been recognized to work as a potent tumor suppressor by controlling cell fate and cell differentiation. The mechanism of AIMP2 as a potent tumor suppressor is related to the triggering of growth-arrest signaling in transforming growth factor-beta, which is caused by the enhancement of the ubiquitin-mediated degradation of the FUSE-binding protein [15]. AIMP2-DX2 interferes with the tumor suppressor activity of AIMP2, and is also overexpressed in lung cancer, which suggests a diagnostic role for lung cancer [16]. With this evidence supporting the possibility of the biomarker of lung cancer diagnosis, we implicate MARS and AIMP2-DX2 staining as a new biomarker in patients with lung nodules on chest CT scans. In this study, we hypothesized that double staining with MARS and AIMP2-DX2 could improve the diagnostic power for lung cancer. However, we found that only MARS staining was suitable as an independent staining method associated with the diagnosis of lung cancer. The combination of AIMP2-DX2 and MARS provided no additional improvement in diagnostic performance. The lack of any diagnostic advantage of double staining was due to the increased expression of AIMP2-DX2 in benign lung nodule cases. An experimental study suggested that the overexpression of AIMP2-DX2 was induced by carcinogenic stress derived from AIMP2, which reduced the pro-apoptotic activity of TNF-α [17]. The overexpression of AIMP2-DX2 in non-malignant cells indicates that inflammation in individual cells could contribute to carcinogenic stress. Further studies are needed to explore the relationship between inflammation and carcinogenetic stress. In addition, the route of lung tissue acquisition could affect the diagnostic performance regarding dual IF. Kim et al. reported the diagnostic performance of MARS, AIMP2-DX2, and Pan-CK in cytology samples obtained by computed tomography-guided needle aspiration biopsy (CT-NAB) [18]. Their results show the discriminative power for cytology samples when dual IF staining is used with any two approaches of MARS, AIMP2-DX2, and pan-CK in combination, compared to conventional cytology alone. MARS plus pan-CK had the best diagnostic yield and pan-CK could be used to differentiate between epithelial- and non-epithelial-oriented cells. However, the pan-CK biomarker is inadequate for use in brushed cytology samples, which contain many bronchial epithelial cells. Furthermore, cellular morphology, including nucleus diameter, presence of nucleoli, and cluster patterns, did not affect the diagnostic power of MARS staining. MARS staining following conventional cytology increased the sensitivity and diagnostic accuracy of lung nodules compared with conventional cytology alone. By contrast, the ROC AUC showed a smaller improvement in diagnostic power than expected. MARS staining increased the sensitivity of conventional cytology from 31% to 70%, and the overall accuracy also increased. However, the specificity of MARS staining decreased to 65% compared with the 100% specificity of conventional cytology. This suggests that conventional cytology could rule out the possibility of malignancy in non-malignant cells better than MARS staining, which showed false positive activity in non-malignant cells.

Although MARS staining showed advantages over conventional cytology, there were several limitations to the present study. First, benign nodules accounted for a relatively small number of cases. Thus, the specificity was not thoroughly evaluated. Second, in addition to the small sample of benign lung nodules, MARS itself may not be able to differentiate benign inflammatory cells from malignant cells. ARSs play roles in both immune and inflammatory processes [17]. For instance, inflammatory cytokines cause the overexpression of tryptophanyl tRNA synthetase (WARS) in infected cells [19]. Previous studies may have assumed that MARS is overexpressed in inflammatory cells, which highlights the need for clarification regarding the relationship between MARS and inflammatory cells in the lungs.

In conclusion, MARS staining as a component of brush cytology achieved greater diagnostic accuracy than MARS and conventional cytology alone. However, further biomarker studies are needed to confirm these findings in large benign samples because of the lower specificity of MARS.

## Figures and Tables

**Table 1:** Baseline patient characteristics.

Test Method	Benign (*n* = 26) No. (%)	Malignant (*n* = 87) No. (%)	*p*-Value
Age, years			0.096
Median	62 (53–70)	66 (59–72)	
Sex			0.804
Male	16 (61.5)	58 (66.7)	
Female	10 (38.5)	29 (33.3)	
Size, mm			0.001
Median	19.5 (14.0–27.0)	28.0 (21.0–36.0)	
Stage			
N/A	26 (100)		
1		41 (47.1)	
2		15 (17.2)	
3		16 (18.4)	
4		14 (16.1)	
LD		1 (1.10)	
Histology	N/A		
Adenocarcinoma		67 (77.0)	
Squamous cell carcinoma		16 (18.4)	
NSCLC, NOS		3 (3.4)	
Small-cell carcinoma		1 (1.1)	
Conventional cytology			0.003
Positive for malignancy	0 (0)	27 (31.0)	
Not malignant	26 (100.0)	60 (69.0)	
MARS staining			0.002
Positive	9 (34.6)	61 (70.1)	
Negative	17 (65.4)	26 (29.9)	
AIMP2-DX2 staining			0.073
Positive	14 (53.8)	65 (74.7)	
Negative	12 (46.2)	22 (25.3)	
Double staining ^a^			0.002
Positive	9 (34.6)	61 (70.1)	
Negative	17 (65.4)	26 (29.9)	

Data are presented as numbers (percentages) and means (ranges) for categorical and continuous variables, respectively. ^a^ MARS and AIMP2-DX2 staining.

**Table 2:** Benign and malignant cytology.

Test Method	Benign (*n* = 26) No. (%)	Malignant (*n* = 87) No. (%)	*p*-Value
Nucleus, µm			
Long diameter	9.8 ± 2.5	11.5 ± 2.7	0.004
Nucleoli			0.190
Absent	13 (50.0)	29 (33.3)	
Present	13 (50.0)	58 (66.7)	
Cluster patterns			0.006
Single cells	8 (30.8)	8 (9.2)	
Linear	3 (11.5)	3 (3.4)	
Three cells in a cluster	3 (11.5)	7 (8.0)	
≥4 cells in a cluster	12 (46.2)	69 (79.3)	

Data are presented as numbers (percentages) and means ± standard deviations for categorical and continuous variables, respectively.

**Table 3:** Unadjusted and adjusted odds ratios (ORs) for the diagnostic results.

	Patients Diagnosed with Malignant Lung Nodules					
	Univariable Analysis			Multivariable Analysis		
	OR	95% CI	*p*-Value	Adjusted OR	95% CI	*p*-Value
MARS	4.43	1.75–11.22	0.002	6.02	1.32–28.09	0.019
AIMP2-DX2	2.53	1.02–6.29	0.045	0.28	0.05–1.50	0.140
Double staining	4.43	1.75–11.22	0.002	NA	NA	NA
Nuclear diameter, μm	1.29	1.08–1.55	0.006	1.25	0.97–1.67	0.416
Nucleoli	2.00	0.82–4.86	0.126	0.56	0.13–2.16	0.655
Cluster pattern						
Single cells	Reference			Reference		
Linear	1.00	0.15–6.53	1.000	0.62	0.07–5.17	0.830
Three cells in a cluster	2.33	0.44–12.40	0.320	1.23	0.19–8.99	0.145
≥4 cells in a cluster	5.75	1.81–18.27	0.003	2.91	0.70–12.81	0.142

**Table 4:** Diagnostic performance of cytology and staining methods.

Test Method	Sensitivity	Specificity	Positive Predictive Value	Negative Predictive Value	Accuracy
Cytology ^a^	0.31	1.00	1.00	0.32	0.48
Anti-DX2 antibody	0.76	0.50	0.82	0.40	0.69
Anti-MARS antibody	0.71	0.68	0.87	0.43	0.70
Double ^b^ antibody	0.71	0.68	0.87	0.43	0.70
MARS + cytology	0.73	0.68	0.88	0.45	0.72

^a^ Conventional cytology with Pap staining; ^b^ Combined staining using anti-MARS and anti-AIMP2-DX2 antibodies.

## Abbreviations

AIMP2-DX2	a variant of Aminoacyl-tRNA synthetase interacting multifunctional protein 2 with an exon 2 deletion
ARSs	aminoacyl-tRNA synthetases
LDCT	low-dose computed tomography
MARS	methionyl-tRNA synthetase
NSCLC	non-small-cell lung carcinoma
R-EBUS	radial endobronchial ultrasound
TBLB	transbronchial lung biopsy

## References

[1] Baaklini W.A., Reinoso M.A., Gorin A.B., Sharafkaneh A., Manian P. (2000). Diagnostic yield of fiberoptic bronchoscopy in evaluating solitary pulmonary nodules. Chest.

[2] Gildea T.R., Mazzone P.J., Karnak D., Meziane M., Mehta A.C. (2006). Electromagnetic navigation diagnostic bronchoscopy: A prospective study. Am. J. Respir. Crit. Care Med..

[3] Anantham D., Koh M.S., Ernst A. (2009). Endobronchial ultrasound. Respir. Med..

[4] Boonsarngsuk V., Kanoksil W., Laungdamerongchai S. (2015). Comparison of diagnostic performances among bronchoscopic sampling techniques in the diagnosis of peripheral pulmonary lesions. J. Thorac. Dis..

[5] Kim S., You S., Hwang D. (2011). Aminoacyl-tRNA synthetases and tumorigenesis: More than housekeeping. Nat. Rev. Cancer.

[6] Han J.M., Jeong S.J., Park M.C., Kim G., Kwon N.H., Kim H.K., Ha S.H., Ryu S.H., Kim S. (2012). Leucyl-tRNA synthetase is an intracellular leucine sensor for the mTORC1-signaling pathway. Cell.

[7] Kushner J.P., Boll D., Quagliana J., Dickman S. (1976). Elevated methionine-tRNA synthetase activity in human colon cancer. Proc. Soc. Exp. Biol. Med..

[8] Marshall L., Kenneth N.S., White R.J. (2008). Elevated tRNA(iMet) synthesis can drive cell proliferation and oncogenic transformation. Cell.

[9] Kim E.Y., Jung J.Y., Kim A., Kim K., Chang Y.S. (2017). Methionyl-tRNA synthetase overexpression is associated with poor clinical outcomes in non-small cell lung cancer. BMC Cancer.

[10] Jung J.Y., Kim E.Y., Kim A., Chang J., Kwon N.H., Moon Y., Kang E.J., Sung J.S., Shim H., Kim S. (2017). Ratio of Autoantibodies of Tumor Suppressor AIMP2 and Its Oncogenic Variant Is Associated with Clinical Outcome in Lung Cancer. J. Cancer.

[11] Reifenberger G., Ichimura K., Reifenberger J., Elkahloun A.G., Meltzer P.S., Collins V.P. (1996). Refined mapping of 12q13-q15 amplicons in human malignant gliomas suggests CDK4/SAS and MDM2 as independent amplification targets. Cancer Res..

[12] Guo M., Yang X.L., Schimmel P. (2010). New functions of aminoacyl-tRNA synthetases beyond translation. Nat. Rev. Mol. Cell Biol..

[13] Ron D., Habener J.F. (1992). CHOP, a novel developmentally regulated nuclear protein that dimerizes with transcription factors C/EBP and LAP and functions as a dominant-negative inhibitor of gene transcription. Genes Dev..

[14] Lee S.W., Cho B.H., Park S.G., Kim S. (2004). Aminoacyl-tRNA synthetase complexes: Beyond translation. J. Cell Sci..

[15] Kim M.J., Park B.J., Kang Y.S., Kim H.J., Park J.H., Kang J.W., Lee S.W., Han J.M., Lee H.W., Kim S. (2003). Downregulation of FUSE-binding protein and c-myc by tRNA synthetase cofactor p38 is required for lung cell differentiation. Nat. Genet..

[16] Choi J.W., Kim D.G., Lee A.E., Kim H.R., Lee J.Y., Kwon N.H., Shin Y.K., Hwang S.K., Chang S.H., Cho M.H. (2011). Cancer-associated splicing variant of tumor suppressor AIMP2/p38: Pathological implication in tumorigenesis. PLoS Genet..

[17] Nie A., Sun B., Fu Z., Yu D. (2019). Roles of aminoacyl-tRNA synthetases in immune regulation and immune diseases. Cell Death Dis..

[18] Kim T., Lee K., Nahm J.H., Kim E.Y., Lee S.H., Chang Y.S. (2021). Methionyl-tRNA synthetase and aminoacyl-tRNA synthetases interacting multi-functional protein-lacking exon 2 as potential diagnostic biomarkers for lung cancer. Am. J. Cancer Res..

[19] Ellis C.N., LaRocque R.C., Uddin T., Krastins B., Mayo-Smith L.M., Sarracino D., Karlsson E.K., Rahman A., Shirin T., Bhuiyan T.R. (2015). Comparative proteomic analysis reveals activation of mucosal innate immune signaling pathways during cholera. Infect. Immun..

